# Greek School Nurses’ Confidence With Diabetes Devices

**DOI:** 10.7759/cureus.65920

**Published:** 2024-08-01

**Authors:** Evangelia Klada, Victoria Alikari, Aikaterini Toska, Maria Saridi, Eleni Albani, Maria Lavdaniti, Sofia Zyga, Evangelos C Fradelos

**Affiliations:** 1 School of Social Sciences, Hellenic Open University, Patras, GRC; 2 Department of Nursing, University of West Attica, Athens, GRC; 3 General Department of Lamia, University of Thessaly, Lamia, GRC; 4 General Department of Lamia, University of Thessaly, Corinth, GRC; 5 Department of Nursing, University of Patras, Patra, GRC; 6 Department of Nursing, International Hellenic University, Thessaloniki, GRC; 7 Department of Nursing, Faculty of Health Sciences, University of Peloponnese, Tripolis, GRC; 8 Laboratory of Clinical Nursing/Department of Nursing, University of Thesssaly, Larissa, GRC

**Keywords:** pediatric diabetes, type 1 diabetes mellitus, school nurses, diabetes devices, confidence

## Abstract

Aim

School nurses often use diabetes mellitus devices as part of the care provided to students with Type 1 Diabetes Mellitus. The aim of this study was to explore the psychometric properties of the Greek version of the Diabetes Devices Confidence Scale (DDCS).

Methods

In this cross-sectional, descriptive study, 143 school nurses completed the DDCS. This is a self-administered questionnaire exploring the nurses’ confidence in the use of diabetes devices. The scale was translated and culturally adapted according to the WHO guidelines. The Intraclass Correlation Coefficient and Cronbach’s Alpha Index were used to explore the reliability and internal consistency, respectively. The construct validity was tested via exploratory and confirmatory factor analysis (EFA, CFA). Data were analyzed via Statistical Package for the Social Sciences (SPSS), version 22.0 (IBM Corp., Armonk, NY, USA).

Results

Significant correlations were observed between the two administrations (p<0.001) indicating the good reliability of the scale (ICC = 0525, p<0.001) while Cronbach’s Alpha was 0.966 suggesting excellent internal consistency. The EFA resulted in a unidimensional solution explaining 53.7% of the total variance. The CFA showed that the model presents good fit to the data.

Conclusions

The DDCS is a reliable and valid tool to test the nurses’ confidence in diabetes devices.

## Introduction

Type 1 Diabetes Mellitus (T1DM), as a chronic autoimmune disease, entails high healthcare costs [1]. The application of new technologies aims to improve glycemic control and quality of life for patients. The understanding of cellular, molecular, and genetic factors associated with T1DM guided the development of devices that improved the diagnosis, treatment, and monitoring of people with T1DM [2]. Devices that provide continuous glucose monitoring systems improve diabetes control and reduce the incidence of hypoglycemia. Hybrid closed-loop solutions, using artificial intelligence algorithms, are a pioneering approach to the management of T1DM. In the future, the development of more sophisticated methods of automated glycemic control is sought [3,4]. Current technologies greatly improve T1DM self-management, while support from family and healthcare professionals enhances quality of life [5].

Managing T1DM in the school is a critical part of care, and the school nurse takes on the role of the student's care provider. This care aims to achieve optimal health for the children and their best academic performance [6]. As students spend approximately 30-35 hours per week in the educational environment, it is vital that children with T1DM feel safe. In such aspects, school nurses must be alert for symptoms of the disease and manage them promptly. In addition, among their responsibilities are monitoring of nutrition status, physical activity, monitoring blood glucose levels, and the management of insulin administration. School nurses also have a crucial role in managing any complications that may arise during school activities [7,8]. They collaborate with parents, teachers, and other members of the school community in order to achieve a safe and healthy school environment for children with T1DM [6-9].

To do so nowadays school nurses often use continuous glucose monitoring systems [10]. When school nurses possess a high level of knowledge and comfort in handling these devices, they are able to effectively monitor blood sugar levels, administer insulin, and promptly respond to any emergencies that may arise. The findings of studies have demonstrated that the knowledge of school nurses in utilizing diabetes devices, such as glucose meters and insulin pumps, significantly impacts the quality of care provided to students with diabetes [11, 12]. Even though such devices are frequently used by nurses often they experience significant stress when using them, which can be due to a variety of factors. The lack of knowledge of the device, technical problems, and time pressure are some of them, while nurses' fear of possible mistakes or harm patients increases anxiety. These stressful situations can negatively affect nurses' performance and patient outcomes [11,13].

Despite the importance of school nurses' role in diabetes device management and their confidence in this area, research in this field is very limited. The lack of research data in this area makes it difficult to understand the level of confidence of nurses and the challenges they may face when using the relevant devices [14]. More research is needed to focus on developing school nurses' confidence in the use and management of diabetes devices. Thus, the aim of this study is to examine Greek school nurses’ confidence in diabetes devices. Moreover, this study aimed to examine the psychometric properties of the Diabetes Device Confidence Scale (DDCS).

## Materials and methods

A cross-sectional study design was employed in this study. Participants were recruited via a Greek group for school nurses on a social media platform. A convenience sample of 143 school nurses with a minimum of one year of school nursing experience participated in this study.

Data collection

Data were collected via an anonymous questionnaire consisting of two parts:

a. The first part contained questions regarding demographic and work-related characteristics such as gender, age, working experience, etc.

b. The second part was the DDCS. The DDCS is a 25-item scale that assesses school nurses’ confidence regarding diabetes devices such as insulin pumps (for example “I can change an insulin pump sit”), continuous glucose monitors, and sensor-integrated insulin pumps. The answers are given on a five-Likert scale ranging from “not at all confident” (1) to “extremely confident.”(5). Thus, the total score ranges from 25-125. The DDCS is a unidimensional instrument and in previous studies demonstrated high reliability (alpha = 0.97) [12,13]. This instrument was first used among school nurses in Pennsylvania [14,15] and, to our knowledge, it has not been used in other studies.

The translation and cultural adaptation process

To translate and culturally adapt the DDCS, the guidelines of WHO [16] were applied. Forward and backward translations were performed to derive the Greek version of the scale. Firstly, the English version was translated into the Greek language by two independent bilingual translators who were health professionals. The two Greek versions were merged into one after editing by a third translator. Subsequently, the Greek version was translated into English by two independent translators who were fluent in English. The two English versions were merged into one by a third translator to produce the English version. The produced Greek version was administered to ten school nurses and the cognitive interview process followed in which school nurses stated whether there were any unclear and difficult-to-understand points. The majority of the school nurses (n = 9) stated that there were no unclear points.

Data analysis

Analyses were performed with the Statistical Package for the Social Sciences (SPSS), version 26.0 (IBM Corp., Armonk, NY, USA), and JASP v.0.14.1.0. The statistical significance was set at <0.05. To describe quantitative variables, mean values, and standard deviations were applied. Absolute and relative frequencies were used to describe qualitative variables. To assess the test-retest reliability between the two measurements, a paired sample t-test was used. To assess the internal consistency reliability of the scale, Cronbach’s Alpha index was applied (accepted values > 0.70) [17]. The construct validity and the factor structure of the DDCS were tested through exploratory and confirmatory factor analysis. Specifically, the following fit statistics were used to explore the model’s global adjustment: the Tucker-Lewis index (TLI), the Root-Mean-Square Error of Approximation (RMSEA[ΒΑ1]), and the Comparative Fit Index (CFI). Values of CFI, TLI ≥ 0.90, and RMSEA ≤ 0.10 indicate an acceptable fit [18]. Correlations between the item scores and the total score of each subscale [ΒΑ2] were applied. Values of the correlation coefficient (r) from 0.1-0.3 indicate low correlation, from 0.31-0.5 moderate, and >0.5 high correlation [19].

Ethics

To conduct the study, licenses were secured from the Research Ethics Committee of the Nursing Department of the University of Thessaly (ND 806.10.06.2022). The school nurses were informed in writing about the scope of the work, the anonymity and confidentiality of the data, that the data would not be shared with anyone, and their right to absent themselves if desired.

## Results

Of the total of 143 school nurses, 61.5% were male, 42.7% were 25-35 years old, the majority were university graduates and had 1-5 years of working experience as school nurses. Bivariate analysis indicated that only educational status among the various demographic and professional characteristics may affect the total score of DDCS. More specifically, results revealed a significant effect of educational status on DDCS, (F(3,139) = 2.893, p < 0.038). Those who hold a Ph.D./M.Sc. degree reported higher scores than those who had a B.Sc. degree. Detailed information regarding the demographic and work-related characteristics as well as bivariate analysis are presented in Table 1.

**Table 1:** Demographic characteristics and bivariate analysis (N=143) DDCS: Diabetes Device Confidence Scale; SD: standard deviation ^a^ For variables with two categories, Independent t-test was used and t-value was calculated; for variables with three or more categories, One-Way ANOVA was used and F value was calculated.

Variables		N	%	DDCS Mean(SD)	Test^a^	P value
Gender	Male	55	38.5	3.97(0.6)	t=1.405	0.162
	Female	88	61.5	3.80(0.7)		
Age	25-35	61	42.7	3.69(0.7)	F=2.113	0.054
	36-45	61	42.7	3.95(0.5)		
	46-55	18	12.6	4.08(0.5)		
	56+	3	2.1	4.38(0.3)		
Education	Ph.D.	9	6.3	4.19(0.4)	F=2.893	0.038
	M.Sc.	59	41.3	3.97(0.7)		
	University Degree	75	52.4	3.74(0.6)		
Working experience as clinical nurses	1-5 Years	107	74.9	3.778	F=1.260	0.291
	6-10 Years	27	18.9	4.056		
	11+ Years	9	6.3	4.031		
Nurses’ School Experience	1-5 Years	94	65.7	3.85(0.6)	F=1.950	0.290
	6-10 Years	48	33.6	4.13(0.7)		
	11+ Years	1	0.7	4.25(0.7)		
Training in Diabetes Mellitus	No	72	50.3	3.80(0.7)	t=-1.107	0.270
	Yes	71	49.7	3.93(0.60)		

The score of the DDCS was found to be 3.87. This value is slightly below the median score thus we can say that Greek school nurses exhibit moderate to high confidence with diabetes devices. The descriptive characteristics of the scale are presented in Table 2.

**Table 2:** Descriptive characteristics of the Diabetes Device Confidence Scale

Mean (SD)	Min	Max	Median
3.87(0.67)	1.32	5.00	4.00
SD: Standard Deviation			

Reliability analysis of the DDCS

The test-retest method was applied to explore the test-retest repeatability of the DDCS. Τwenty-five school nurses completed the questionnaire at baseline and two weeks later. This interval is interposed so that the individuals do not recall their answers [16]. Upon the analysis, significant correlations were observed between the two-administration (p<0.001) facts (Intraclass Correlation Coefficients = 0.525) that reveal that the scale is stable through time.

In addition, Cronbach’s Alpha had a value of 0.966 suggesting excellent internal consistency of the scale (Table 3). Moreover, the value of Cronbach’s Alpha didn’t increase if items were discarded by the scale. All items exhibited strong correlations to the total score. This fact adds to the excellent internal consistency of the scale.

**Table 3:** Exploratory Factor Analysis and Reliability Analysis

Items	Factor Loadings	Uniqueness	Cronbach’s Alpha if item deleted	Item to total correlation
q1	0.783	0.387	0.964	0.769
q2	0.739	0.455	0.964	0.721
q3	0.697	0.515	0.965	0.688
q4	0.798	0.363	0.964	0.782
q5	0.759	0.424	0.964	0.750
q6	0.744	0.447	0.964	0.734
q7	0.743	0.448	0.964	0.731
q8	0.682	0.535	0.965	0.672
q9	0.756	0.429	0.964	0.744
q10	0.709	0.497	0.965	0.696
q11	0.772	0.404	0.964	0.760
q12	0.753	0.433	0.964	0.738
q13	0.755	0.429	0.964	0.737
q14	0.804	0.353	0.964	0.792
q15	0.616	0.620	0.965	0.609
q16	0.537	0.712	0.966	0.527
q17	0.589	0.653	0.966	0.579
q18	0.765	0.415	0.964	0.752
q19	0.820	0.328	0.964	0.805
q20	0.674	0.546	0.965	0.658
q21	0.648	0.580	0.965	0.636
q22	0.773	0.402	0.964	0.756
q23	0.777	0.396	0.964	0.764
q24	0.803	0.355	0.964	0.790
q25	0.746	0.443	0.964	0.729
Cronbach’s alpha = 0.966				
Intraclass Correlation Coefficients (ICC) = 0.525, p<0.001				
Bartlett's test Χ² = 2.738.408, Df= 300.000 p				
Total variance explained 53.7%				

Exploratory and confirmatory factor analyses were applied to examine the factorial structure of the DDCS. In the first step, the Kaiser-Meyer-Olkin (KMO) statistic and Bartlett’s sphericity test were performed to examine the sample’s adequacy for factor analysis. The value of x2 and the significance of the Bartlett test (p<0.001) indicated that data are adequate for factor analysis. Exploratory factor analysis was performed with principal component analysis to identify items’ factors. The appropriate number of factors was determined by eigenvalues greater than 1. The EFA resulted in a unidimensional solution for the scale interpreting 53.7% of the variance, a fact that is in line with the original version (Table 3).

Construct validity of DDCS

Finally, we performed a CFA to test the one-factor structure of the scale. Regarding CFA, the model tested was equivalent to the original factorial structure of the DDCS as proposed by the authors. The model presented a reasonably good fit to the data. The chi-square/df ratio was 2.24, less than 3.00 indicating an acceptable fit, Tucker-Lewis index (TLI) was near 0.8, comparative fit index (CFI) was close to 0.9 and standardized root mean square residual (SRMR) was 0.057 and lower than 0.10. Overall, our CFA confirmed the unidimensional structure of the scale.

## Discussion

The present study aimed to assess Greek school nurses’ confidence in diabetes devices and to examine the psychometric properties and factorial structure of the Greek version of the DDCS.

The DDCS has not been used in other countries except for the first study in Pennsylvania so there are no more studies to compare our results. The translation and cultural adaptation of a questionnaire is a complex issue. It involves adjusting the scale to accommodate the specific cultural elements of each country. To achieve this, the method of forward and backward translation was employed according to the WHO guidelines [15], involving the collaboration of four independent translators. These translators were highly skilled health professionals who demonstrated a proficient command of the English language. The Greek version of DDCS was favorably received by Greek school nurses in school settings, as they found the questions to be easily comprehensible. The 10-minute timeframe required to complete the questionnaire was considered acceptable.

The results of the repeatability test revealed that there were no significant variations in the ICC values between the first and second measurements, signifying the stability of the characteristic being assessed. The Cronbach’s alpha coefficient of the total score of the Greek version was 0.966. The Cronbach’s alpha coefficient reported in our research closely resembles those of the original scale (Cronbach’s a 0.97), confirming a high level of internal consistency [16]. This suggests that the scale is a reliable tool that can assess the confidence that Greek nurses must operate and manage diabetes devices. In addition, the exclusion of an item from the scale did not cause any change in the Cronbach's Alpha Index. This outcome indicates that all the items displayed excellent internal consistency in relation to each other.

As far as the construct validity is concerned, the DDCS’s unidimensionality was confirmed through EFA, which produced a single solution accounting for 53.7% of the total variance. This finding aligns with the original version of the scale, indicating consistency in its unidimensional nature. All item loadings were found to be high with most items demonstrating higher loadings in the current study (0.537-0.820) [16].

Diabetes devices are very important in daily clinical practice, providing diabetes patients with the means to monitor and control their blood glucose levels. These devices include glucometers, continuous glucose monitors, and insulin pumps. The use of these devices allows patients to maintain stable blood glucose levels, preventing complications and improving their quality of life [20]. Advances in the field of molecular biology had an important role in the field of diabetes therapy and management. Understanding the molecular mechanisms that regulate cell growth, genetic diseases, and immunological processes has enabled the development of new diagnostic methods and therapies in diabetes but also in many diseases. Molecular biology also provides the basis for the development of drugs that target specific biological processes, thereby improving the effectiveness and precision of treatment [21].

Overall, the role of school nurses in the management of students with T1DM is assessed as important, and appropriate education contributes decisively to this but, also, the feeling of self-confidence on their part. In fact, there are studies that have highlighted this role [22]. The daily presence of a school nurse reduces absences, significantly improves school performance, and enhances diabetic management of students with T1DM. In addition, the teachers also feel safer with the presence of a school nurse according to a study carried out in Greece [22].

The entry of new technologies into the health sector opens new horizons for the improvement of the health system. Lifelong and continuous education of nurses in these technologies is a critical element in upgrading the nursing profession, as well as in offering quality treatment to patients and diseases. The successful use of technology has resulted in saving time and money, as well as providing life-saving interventions in the course of diseases [23]. Many healthcare services including school nursing can benefit from the integration of new technology, supporting cases that require special care and expertise. Investment in equipment and continuous training of nursing staff are essential tools for the effective management of critical situations and the smooth operation of clinics [24].

According to our results, Greek school nurses exhibited moderate confidence in using diabetes devices. Nurses’ competencies in the use of digital technologies have been examined in other studies in various healthcare settings. According to an Australian study that examined the use of IT by nurses and the barriers to this using a self-administered questionnaire, 86% of respondents used computers at work, with the most common uses being patient record management, continuing professional education, communication, accessing policies and procedures, and accessing clinical results. However, experience and confidence in using technology were generally low, with less than 25% of nurses reporting that they were very experienced in using any software application. Results vary depending on the nurse's level, age, and length of time in the nursing profession. The researchers suggested that employers and policymakers at all levels of government must work with nurses to adopt strategies that will increase their access to IT and remove barriers to its use [25,26]. Similarly, a study [27] that examined nurses' perceptions of and confidence in the use of the electronic medical record system in hospital-based healthcare settings, reported that the majority of participants had no prior experience with electronic medical record systems elsewhere. Approximately 50% of the participants indicated that they possessed a sense of assurance when utilizing the system. According to a logistic regression model, nurses with at least six years of experience utilizing the system reported that their recommendations for enhancing the system were acknowledged by the management team. Furthermore, they emphasized the importance of the changes introduced to the system in relation to their work and expressed confidence in the system's ability to provide up-to-date information. These findings suggest that nurses with greater experience are more likely to exhibit confidence in utilizing the system. In order to enhance the readiness of upcoming nurses to adopt contemporary technology-led healthcare practices, it is advisable to incorporate comprehensive IT education into nursing curricula [27].

Strengths and limitations

The strength of this study lies in its focus on a relatively unexplored area: the confidence that school nurses in Greece have in managing diabetes devices. However, the study is not without its limitations. Firstly, its cross-sectional design restricts the ability to comprehend the longitudinal changes in the confidence levels of school nurses regarding the management of diabetes devices. Secondly, the study's small sample size limits the generalizability of the findings. Finally, more studies need to be carried out to examine and fully comprehend nurses' confidence in using diabetes devices. Without additional studies using DDCS for comparison, the generalizability of our findings is limited. This lack of comparative data makes it challenging to determine whether our results are consistent across different populations and settings.

## Conclusions

This study provides some evidence regarding the level of confidence of Greek school nurses in the use of devices for diabetes management. Results indicate that nurses have moderate to high confidence in diabetes devices, with a mean DDCS score of 3.87 and a median of 4.00. In addition, nurses with higher education, such as a master's or PhD, have significantly higher confidence in using these devices, which highlights the importance of advanced education and continuing professional development. This is critical to effectively supporting students with diabetes in schools.

The DDCS scale was shown to have excellent psychometric properties, with a high Cronbach's Alpha value (0.966), indicating excellent internal consistency. The result of the factor analysis confirms that the scale is unidimensional and explains a significant proportion of the variance in confidence in the use of diabetes devices. The stability of the scale over time and the strong correlations of the individual questions with the total score support the application of the DDCS in different contexts and populations, facilitating the assessment of nurses' confidence. Overall, this study makes a significant contribution to the understanding of school nurses' confidence in using diabetes devices and supports the use of the DDCS to enhance this confidence, which is critical to providing quality care to students with diabetes.
